# Why the Immune System Should Be Concerned by Nanomaterials?

**DOI:** 10.3389/fimmu.2017.00544

**Published:** 2017-05-15

**Authors:** Marc J. Pallardy, Isabelle Turbica, Armelle Biola-Vidamment

**Affiliations:** ^1^“Inflammation, Chimiokines and Immunopathology”, INSERM UMR 996, Univ Paris-Sud, Université Paris-Saclay, Châtenay-Malabry, France

**Keywords:** nanoparticles, dendritic cells, danger signals, macrophages, innate immunity

## Abstract

Particles possess huge specific surface area and therefore nanomaterials exhibit unique characteristics, such as special physical properties and chemical hyper-reactivity, which make them particularly attractive but also raise numerous questions concerning their safety. Interactions of nanomaterials with the immune system can potentially lead to immunosuppression, hypersensitivity (allergy), immunogenicity and autoimmunity, involving both innate and adaptive immune responses. Inherent physical and chemical NP characteristics may influence their immunotoxicity, i.e., the adverse effects that can result from exposure. This review will focus on the possible interaction of nanomaterials including protein aggregates with the innate immune system with specific emphasis on antigen-presenting cells, i.e., dendritic cells, macrophages and monocytes.

## Introduction

Nanoparticles (NP) are defined as structures with at least one dimension in the range of 1–100 nm. At this nanoscale, particles possess huge specific surface area. Nanomaterials therefore exhibit unique characteristics, such as special physical properties and chemical hyper-reactivity, which make them particularly attractive but also raise numerous questions concerning their safety. Nanomaterial interactions with the body include accidental exposure (environmental and industrial NP) and therapeutic exposure (vaccination, drug delivery). Virtually, all the possible routes of exposure (inhalation, ingestion, dermal contact, systemic injection) have to be considered.

The main objective of the immune system is to avoid harmful effects due to contamination by microbes and also to maintain an immune tolerance to environmental antigens. To distinguish between harmful and non-harmful antigens, the dendritic cells (DCs) play a major role by sensing the environment and adapting their phenotype to the most appropriate type of response: immunogenic vs. tolerogenic. Interactions of NP with the immune system can potentially lead to immunosuppression, hypersensitivity (allergy), immunogenicity and autoimmunity, involving both innate and adaptive immune responses. Inherent physical and chemical NP characteristics may influence their immunotoxicity, i.e., the adverse effects that can result from exposure. This review will focus on the possible interaction of nanomaterials, including protein aggregates, with the innate immune system with specific emphasis on antigen-presenting cells, i.e., DCs, macrophages and monocytes.

## NP Interaction with Innate Immune Cells

In host, the mononuclear phagocytic system plays a major role in the exposure to nanomaterials. Macrophages are in charge of nanomaterials recognition, uptake, processing, and clearance (1). Several *in vivo* studies have demonstrated high NPs macrophage sequestration, particularly in clearance organs such as liver, spleen, and kidney. In these organs, fenestrated capillary beds, competent to capture particles, are associated with specialized macrophages populations (1). In mice injected with non-degradable silica NPs, a high accumulation in the liver and in the spleen was observed, in majority in the macrophages but also in neutrophils (2). This property could be responsible for organ-specific toxicity, especially in the liver, of some NPs.

Nanoparticles uptake can occur through phagocytosis, macropinocytosis, as well as clathrin-, caveolae-, and scavenger receptor-mediated endocytic pathways. These internalization processes are deeply dependent on nanomaterials properties such as size, shape, surface coating, and on the cellular environment (3). Phagocytosis is carried out by professional phagocytes such as macrophages, neutrophils, DCs, or monocytes. Due to their actin-based cytoskeleton rearrangement capacities, these cells can entrap the material through membrane dynamics in a zipper model fashion (1). The best characterized opsonin-dependent phagocytosis receptors are the Fcγ receptor and the complement receptor CR3, which appear to play a significant role in the detection of opsonized nanomaterials and in the rate of uptake (1). It was demonstrated that the small gold colloid NPs (30 nm) use several internalization routes (including scavenger receptor-, clathrin-, and caveolin-mediated pathways), in contrast to the larger materials of 150 nm which appear to be preferentially taken up via the scavenger receptor pathway (4). The scavenger receptor MARCO has been involved in the ingestion of unopsonized inhaled TiO_2_ and Fe_2_O_3_ particles in the lung (5). Moreover, the recognition of silica NPs by macrophages scavenger A receptor could induce the release of cytokines responsible for pulmonary inflammation (6). The mechanisms for NP uptake by DCs are poorly understood. However, according to Vallhov et al. (7), an active mechanism such as endocytosis may be involved in the amorphous silica nanoparticle (aSNP) uptake by DCs (7). Winter et al. (8) additionally suggested that it would be at least partly mediated by an actin-dependent mechanism (8).

Nanomaterials can affect the polarization and the reprogramming of macrophages, mostly depending on chemical composition, size, and surface modification (9). The pro-inflammatory M1 or anti-inflammatory M2 phenotypes have been shown to display distinct uptake capacity for nanomaterials. In particular, silica NPs uptake is enhanced in M2-polarized primary human monocyte-derived macrophages or in the macrophage-like THP-1 cell line as compared with M1 cells (10).

*In vivo*, upon exposure to biological fluids, NPs do not stay “naked” but become coated by biomolecules, primarily proteins but also sugars, lipids, or nucleic acids, forming a “corona” (11). This corona is “what the cell sees” and displays a highly dynamic nature: changes in the composition occur over time, in a continuous flux of desorption/adsorption of proteins. If the “hard” corona is tightly bound with a long exchange time, the “soft” corona, presented as a second layer, is submitted to fast exchanges (12, 13). Interestingly, this process could be compared to the opsonization of pathogens (14) and affects the efficiency of NPs uptake by macrophages. Kapralov et al. demonstrated that single-walled carbon nanotubes (SWCNTs) selectively adsorbed phosphatidylcholines and phosphatidylglycerols from lung surfactant. The presence of this coating noticeably enhanced the *in vitro* uptake of SWCNTs by macrophages (15). Moreover, proteins may undergo conformational changes, such as unfolding, leading to the possible exposition of cryptic epitopes recognized by immune cells (14). This unfolding was demonstrated with fibrinogen coated on negatively charged poly(acrylic acid) gold NPs, leading to MAC-1 receptor activation and pro-inflammatory cytokines secretion through NF-κB signaling (16). Interestingly, only the negatively charged NPs induced TNF-α and IL-8 release by THP-1 cells, whereas both positively and negatively charged particles could bind fibrinogen with high affinity (17). This protein corona is essential for scavenger receptor-efficient internalization of synthetic-layered silicate NPs by THP-1 cells (18). When bound to these NPs, albumin undergoes unfolding, comparable to heat denaturation, revealing a cryptic sequence allowing recognition of serum albumin by this family of receptors and nanomaterial recognition by macrophages (18).

## DCs and Nanomaterials as Exogenous Danger Signals

Dendritic cells are professional antigen-presenting cells that bridge the innate and adaptive immune response. Immature DCs reside in non-lymphoid tissues in an antigen-capturing state. In the presence of various stimuli, such as allergens, inflammation, pro-inflammatory cytokines, bacterial products, or diverse danger signals, DCs undergo a maturation process. This process results in antigen-processing and upregulation of major histocompatibility complex (MHC), co-stimulatory molecules, chemokine, and cytokine receptors, and production of cytokines and chemokines. Mature DCs then migrate to regional lymph nodes and activate naïve T-lymphocytes. Consequently, NP impact on these cells raises growing concerns.

The size of the NP may determine the modulation of DC functions. For example, *in vivo*, 20 nm polystyrene (PS) particles are more frequently captured by lung DCs than 1,000 nm PS particles (19). If the 20 nm PS particle *in vitro* treatment did not affect murine bone marrow-derived dendritic cells (BM-DCs) cell viability, maturation markers expression, and antigen uptake, these particles significantly downregulated antigen degradation in a size-dependent manner, in association with accumulation in lysosomes but without altering T-cell proliferation (19). Moreover, NPs and materials traffic to the draining lymph nodes also appear to be size-dependent. Indeed, only small particles (20–200 nm) are able to drain freely to the lymph nodes (20).

**Figure 1:**
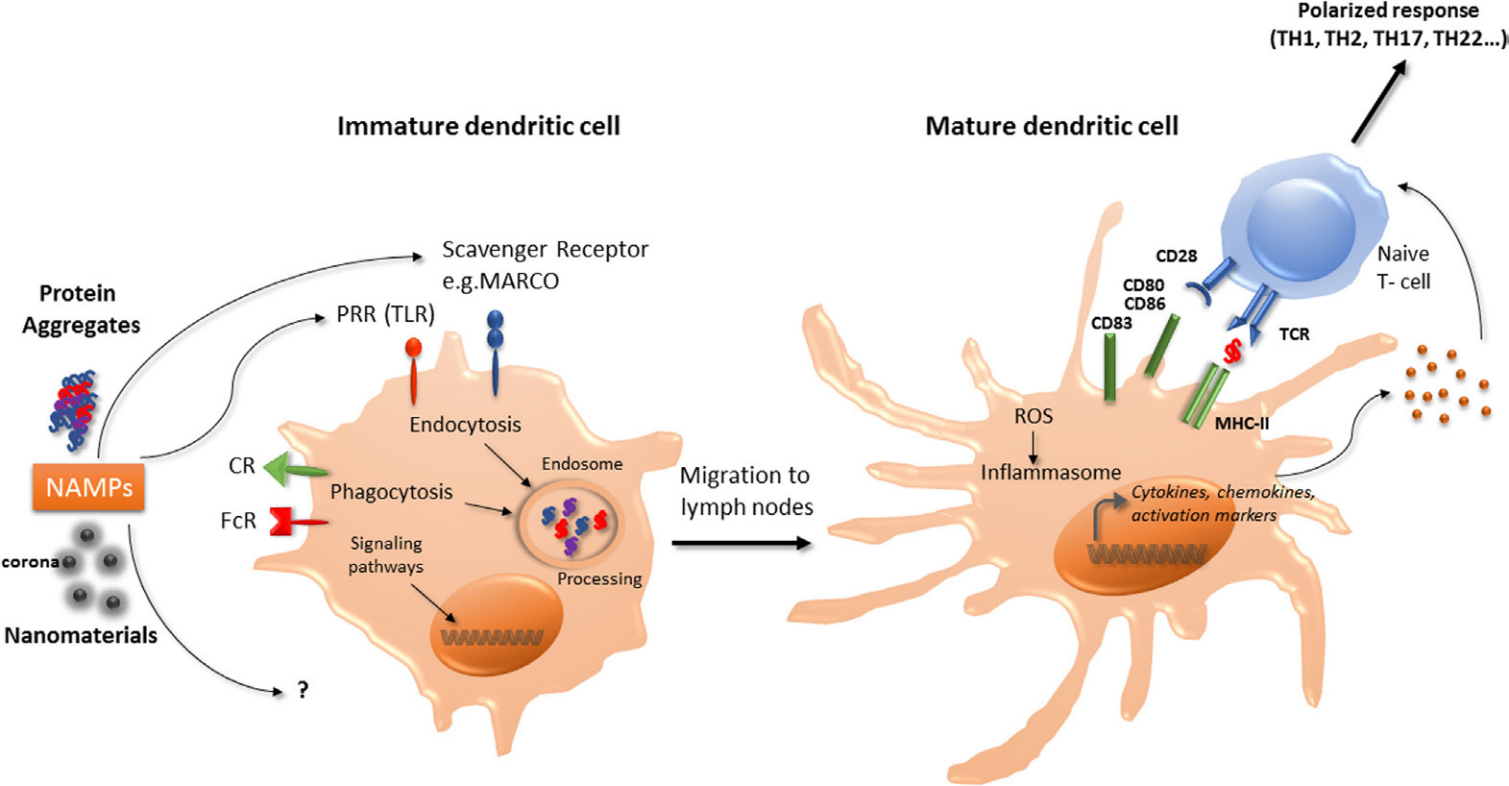
**Interaction of nanomaterials and aggregates with DCs**. Nanomaterials and aggregates can be internalized by several receptors present at immature DCs membrane, either by endocytic or phagocytic pathways. Protein aggregates will then be processed by DCs, leading to peptide presentation associated with MHC class II molecules to naive T-lymphocytes. Both nanomaterials coated with a corona or protein aggregates may also be seen as NAMPs and interact with PRR. This interaction can act as a danger signal that induces a signaling cascade leading to the transcription of maturation genes. Mature DC will then be able to express co-stimulation molecules and to produce cytokines and chemokines that will trigger naïve T-cells activation and polarization. These products can also increase ROS production and initiate the inflammasome activation. CR, complement receptor; DCs, dendritic cells; FcR, immunoglobulin constant fragment receptor; MHC, major histocompatibility complex; NAMP, nanoparticles-associated molecular patterns; PRR, pattern recognition receptors; ROS, reactive oxygen species; Scavenger R, scavenger receptor; TLR, toll-like receptor.

In murine BM-DCs, carbon black NPs upregulate the expression of the cell surface molecules CD86, and slightly CD80 and MHC-II molecules, associated with enhancement of allogenic-mixed lymphocyte reaction (21). TiO_2_ NPs were also demonstrated to increase the expression of CD86, CD80, MHC-II, and TNF-α in murine BM-DCs (22). In murine BM-DCs and in the murine DC line DC 2.4, ultrafine silica NPs decreased cell viability, induced slight phenotypic changes but significantly increased TNF-α production in a size-dependent manner (23). Interestingly, these effects were correlated with inflammatory response *in vivo* in C57BL/6 mice injected subcutaneously with liquid matrigel containing silica NPs (23). Winter et al. (8) studied the effects of aSNPs on murine BM-DCs. Amorphous SNPs were able to affect cell viability through apoptosis and induced partial maturation of BM-DCs as evidenced by enhanced expression of MHC-II and co-stimulatory molecules at the cell surface. Activation of the NLRP3 inflammasome was also reported (8). Taken together, these observations suggest that certain NP may promote DC maturation and activation, thereby leading to T-lymphocytes activation (Figure 1).

## The “Danger Hypothesis” Applied to Exogenous Particles and Nanomaterials

Danger signals of endogenous or exogenous origin activate DCs and stimulate both the innate and adaptative immune responses. As proposed by Gallo and Gallucci, “classic,” “homeostatic,” and “emerging” danger signals can be distinguished (24). Classic danger signals are derived from pathogens and released during infections (pathogen-associated molecular patterns) or result from tissue damage, released by necrotic dying cells (damage-associated molecular patterns or “alarmins”) (25). Homeostatic danger signals are endogenous molecules released during cellular stresses such as hypoxia, acidity, or osmolality perturbations. Chemical sensitizers involved in contact allergy have recently being found to modify the cutaneous microenvironment and/or directly activate DCs resulting in DC phenotype modifications necessary for immune sensitization to these chemicals (26). Emerging danger signals are newly man-made materials, including nanomaterials, and may either directly activate DCs or indirectly by inducing tissue damage. Thus, it is postulated that immune cells could sense nanomaterials, which could be designated as nanoparticles-associated molecular patterns (Figure 1) as described for pathogens (14, 24). Sensing of damage signals can be associated with the constitution of inflammasomes, acting as a multiprotein platform to activate caspase-1 and to stimulate the processing of pro-IL-1β. An increase in reactive oxygen species (ROS) production by nanomaterials has been described as an initiating step in the activation of the inflammasome. Interestingly, TiO_2_ NPs, associated with the generation of ROS in human DCs, promoted cells maturation and pro-inflammatory cytokine release, whereas CeO_2_ NPs, possessing antioxidant properties, triggered human DCs toward an anti-inflammatory profile with IL-10 production (27). Inflammasome activation can also occur through destabilization and rupture of the lysosome following phagocytosis. Indeed, the lysosome compartment is the most described intracellular site of NP sequestration following endocytosis (28). Morishige et al. (29) demonstrated in THP-1 cells that aSNP could induce ROS production, triggered endosomal rupture followed by the activation of NLRP3 inflammasome, and subsequent IL-1-β production (29). These authors therefore established a direct relationship between oxidative stress and IL-1-β secretion. Nano TiO_2_ and nano SiO_2_ particles activate the NLRP3 inflammasome in THP-1 cells, correlated with induction of lung inflammation *in vivo* requiring IL-1 receptor expression (30). Inflammasome activation by nano TiO_2_ and nano SiO_2_ particles would occur through ATP release and adenosine receptor signaling (30, 31). Moreover, 30 nm silica NPs can induce intracellular ATP release and P2X7 receptors purinergic signaling, leading to ROS production, inflammasome activation and stimulating the production of IL-1β and IL-18 in LPS-matured murine BM-DC (32).

## Protein Aggregates, as NPs, Can Drive Immune Responses

Beyond the strict definition of NPs, we should also consider nanomaterials in a broader sense of the term, since other structures than those derived from nanotechnologies could interact with the immune system (33). The example of protein aggregates is deeply studied as therapeutic bioproducts (BP) have a propensity to form oligomeric structures that could be assimilated to NPs. It is now well accepted that aggregation of therapeutic proteins is associated with increased potential for immunogenicity in patients, leading to the development of anti-drug antibodies (34, 35). While the aggregation process is strictly followed and controlled during BP manufacturing process, using orthogonal analysis methods (36), this is no more the case over transportation, storage, and administration procedures. Several studies have shown that under accelerated stress conditions, proteins can give mixtures of soluble aggregates that are submicron species including oligomers or multimers, mostly detected with dynamic light scattering method, and insoluble aggregates that are above the micrometer range (37). This was the case for human growth hormone submitted to a stir stress that gave homogenous aggregates around 892 nm (38), or antibody preparations that underwent stir stress (39), or thermal stress (40, 41). Another study showed the appearance of nanosized antibody aggregates upon heat or pH-shift stress that persisted when preparations were diluted in human serum, highlighting the interactions of aggregated proteins with biological fluids (42). A classification scheme was proposed for antibodies aggregates, based on several biophysical characterizations, in which nanosized particles were present in most of the depicted classes (43), although they were more represented in the subclass showing “small, partially folded and partially reversible” aggregates (43). Moreover, protein aggregation can be promoted by the presence of some other nanosized particles, such as glass (44), tungsten (45), or leaching from vial stoppers, as hypothesized in the early 2000s, regarding the episode of increased pure red-cell aplasia cases in patients treated with epoietin alpha (46). Such cases were shown to be mediated by anti-erythropoietin antibodies cross-reacting with the endogenous protein. Several models highlighting protein interactions and aggregation promoted by shedding particles from administration materials have been described (47–49).

The effect of protein aggregates on the immune system can be evaluated using *in vivo* models, such as immune-tolerant transgenic mice that can be treated with the human native or aggregated recombinant protein. Immunogenicity is then assessed following IgG titers developed against the administrated component. Such transgenic mice models have been developed for interferons (50, 51), and a recent paper showed that recombinant interferon beta aggregates induced a break of immune tolerance in transgenic mice, related with the size and structure of the generated aggregates (52). Using a conventional murine model, another study highlighted that oligomeric antibody aggregates were more immunogenic than larger highly aggregated particles (41), suggesting that protein aggregation that maintains some native epitopes is more immunogenic. However, the use of *in vitro* models is more convenient to test the effect of aggregated proteins on immune cells. Thus, antibody aggregates have the potential to increase the production of inflammatory cytokines by human PBMC (53). Testing these aggregates by size showed that nanosized particles induced a lower response than micro-sized particles (54). The current hypothesis is that aggregates could behave as danger signals and may have mainly an effect on antigen-presenting cells, such as monocytes or DC (Figure 1). This hypothesis was objectivized demonstrating that aggregates interaction with PBMC or primary monocytes is partly mediated by toll-like receptors (TLR2 and TLR4), although other receptors such as Fc or complement receptors are also involved (53, 55). DCs are innate immune cells in first line upon therapeutic protein administration, either by intramuscular, intravenous, or subcutaneous administration, as proteins and aggregates rapidly transit in lymph nodes and interact with resident DCs. Also, cutaneous DCs that are present in the point of injection area could be recruited and migrate to peripheral lymph nodes (56). As therapeutic proteins can be processed by DCs to be presented to T cells, aggregates can interact with pattern recognition receptors, and then induce DCs activation. Indeed, several studies have shown that antibodies or growth hormone (GH) aggregates have the capacity to induce monocyte-derived dendritic cells maturation, evidenced by an increase in phenotypic markers expression, as well as cytokine or chemokine production (38, 57, 58). Both GH and antibodies aggregates could induce the production of IL-6, IL-8, IL-12p40, and CXCL10 whereas CCL2, CCL3, CCL4 production was only seen with GH aggregates (38). These observations could be extended using the monocytic cell line THP-1, that secreted inflammatory cytokines upon incubation with aggregated intravenous immunoglobulin preparations (55). Antibody aggregates are able to induce an increase in CD4+ T-cell proliferation and to drive T-cell polarization, compared to native counterparts through DCs phenotype modifications (38, 53, 57, 58). Cellular mechanisms by which protein aggregates induce DCs maturation remain to be clarified; however, a few elements are available. It was determined that DCs in contact with aggregates presented a higher number and different class II HLA-associated peptides than native counterparts, suggesting different processing and presentation, and thus neo-epitopes presentation (57). Although internalization in DCs lysosomal compartment of aggregated antibodies has been evidenced (58), the exact mechanism, either phagocytosis or macropinocytosis remains to be elucidated. Both certainly take place, depending on the size of the particles (20, 59, 60).

## Conclusion

Why the immune system should be concerned by nanomaterials? From the literature, it is now clear that exposure to environmental particles can exacerbate or participate to allergic manifestations such as asthma or rhinitis. Diesel exhaust particles and, more recently, products generated through the use of nanotechnology have been shown to have detrimental effects on the respiratory systems, with an exacerbation rate of asthma (61). Nanomaterials can alter *in vitro* and *in vivo* responses of the immune system to allergens and can also play a role in allergen sensitization. Mimicking danger signals can lead to a direct effect of DCs phenotype (Figure 1) having consequences on the adaptive immune system response and recognition of allergens. The recent advances in nanotechnology could also lead to unforeseen adverse health effects mediated by the immune system, nanoimmunosafety, in exposed human subjects (62).

## Author Contributions

MP organized the manuscript and wrote the general part. AB-V wrote the nanomaterial part of the article. IT wrote the “aggregates” part of the article.

## Conflict of Interest Statement

The authors declare that the research was conducted in the absence of any commercial or financial relationships that could be construed as a potential conflict of interest.
